# Complex System Analysis of Korean Peninsula Earthquake Data

**DOI:** 10.1038/s41598-020-59619-7

**Published:** 2020-02-14

**Authors:** Sangwon Chae, Suyoung Jang, Sangmok Lee, Donghyun Lee

**Affiliations:** 1 Korea Polytechnic University, Department of Business Administration, Siheung, 15073 Republic of Korea; 2 Mesh Korea, Data Science Team, Seoul, 06193 Republic of Korea

**Keywords:** Environmental impact, Natural hazards

## Abstract

Earthquakes are natural disasters that cause damage in a wide range of regions and represent a complex system that does not have a clear causal relationship with specific observable factors. This research analyzes the earthquake activities on the Korean Peninsula with respect to spatial and temporal factors. Using logarithmic regression analysis, we showed that the relationship between the location of the earthquake and its frequency in these locations follows a power law distribution. In addition, we showed that since 1998 the average earthquake magnitude has decreased from 3.0143 to 2.5433 and the frequency has risen by 3.98 times. Finally, the spatial analysis revealed significantly concentrated earthquake activities in a few particular areas and showed that earthquake occurrence points have shifted southeast. This research showed the change in earthquake dynamics and concentration of earthquake activities in particular regions over time. This finding implies the necessity of further research on spatially-derived earthquake policies on the change of earthquake dynamics.

## Introduction

Damage caused by earthquakes is difficult to predict^1^ because they are a complex system that cause new phenomena and disorder through interaction between its components^2–4^. This complex system can be found in both social^5–7^ and natural^8–11^ phenomena, and there are studies analyzing such complex systems using the power law^12–15^ and complex networks^16–21^. Therefore, to investigate the earthquake phenomena of the Korean Peninsula, this study examines the change of earthquake patterns with time and the distribution of the power law according to space and magnitude.

The selected region for this research, the Korean Peninsula, is located in the stable intraplate region of East Asia^22^. With an average of 41.65 earthquakes stronger than ${M}_{L}$2 by year, it is considered a relatively safe area in terms of earthquakes^23^. However, in 2016 and 2017, the number of earthquakes stronger than ${M}_{L}$2 in the Korean Peninsula, was 254 and 223, respectively. Moreover, on September 12th, 2016, an ${M}_{L}$5.8 earthquake occurred in Gyeongju, Gyeongsang-do Province, being recorded as the strongest earthquake so far. On November 15th, 2017, an ${M}_{L}$5.4 earthquake occurred in Pohang, Gyeongsang-do Province, the second strongest earthquake in Korea. They caused the greatest social and economic damage compared to all past earthquakes in the Korean Peninsula^24^. These consecutive strong earthquakes have increased public interest in earthquakes^25^.

Various earthquake studies have been conducted to analyze their complexity. Omori validated the power law distribution between earthquakes^26^ and their aftershocks, and Gutenberg proved that if the earthquake magnitude is small, the frequency increases in a constant ratio^27^. Using identified earthquake power laws from previous studies, Serra and Corral compared the truncated gamma and tapered Gutenberg–Richter distributions to the simple Gutenberg–Richter power law distribution. They proved that truncated gamma distribution shows significant improvement over the simple power law when fitting the earthquake moment distribution of the whole Earth^28^.

Spatial factors are the key elements to understanding the original form of the earthquake, the damage caused by the earthquake, and earthquake-related social policymaking^29^. Liu conducted research on the spatiotemporal complexity of earthquakes by applying a complex network to sections divided according to rock mass, which are based on earthquake and ground mass data. As a result, the connectivity distribution of the earthquake network showed a power law distribution between the average maximum magnitude of a region and frequency^30^. Earthquake networks have also been used to consider the spatial factors of earthquakes^31–33^. Moreover, previous studies have analyzed earthquake networks based on their sequence^34,35^, and Abe concluded that earthquake studies based on time distribution were necessary, using data from different regions^36^.

In Korea, studies are focused around Gyeongju and Pohang, where the largest earthquake occurred so far^37–39^. In particular, Kim and Choi studied active faults, concluding that further studies are needed to produce active fault maps^40,41^. According to a study of the Yangsan fault zone, which resulted in the largest earthquake on the Korean Peninsula, four significant deformation episodes occurred in the fault, and the dextral slip faulting in the late Paleogene had a significant effect on the deformation. Due to the deformation of the Yangsan fault, it was temporarily reactivated^42^.

This research considers spatiotemporal perspectives. We identified the relationship between the spatial-location and the frequency of earthquakes and its activities in space, along with an analysis of earthquake activity changes according to time. Spatial data was created based on the longitude and latitude of earthquakes that occurred in the Korean Peninsula. While previous studies divided the space into equal areas based on the rock mass, this study creates rectangular grid-sections based on the extreme latitude and longitude points of the earthquake. An analysis of the relationship between the grid-sections and the frequency of earthquakes in these grid-sections is conducted. In addition, the Gutenberg–Richter law is established between the magnitude and frequency of earthquakes by period, and the change in earthquake activity by period is identified to assess the overall change of earthquake activity within the Korean Peninsula.

## Results

### Analysis of earthquake activities in the Korean Peninsula considering spatial factors

First, to verify that the earthquakes are intensively concentrated in a few areas, the Korean Peninsula was equally divided using a 40 × 40 uniform grid forming 1,600 spatial reference points. Then, the frequency of earthquakes per grid-section and the distribution of each grid-section was verified using logarithmic regression.

**Figure 1:**
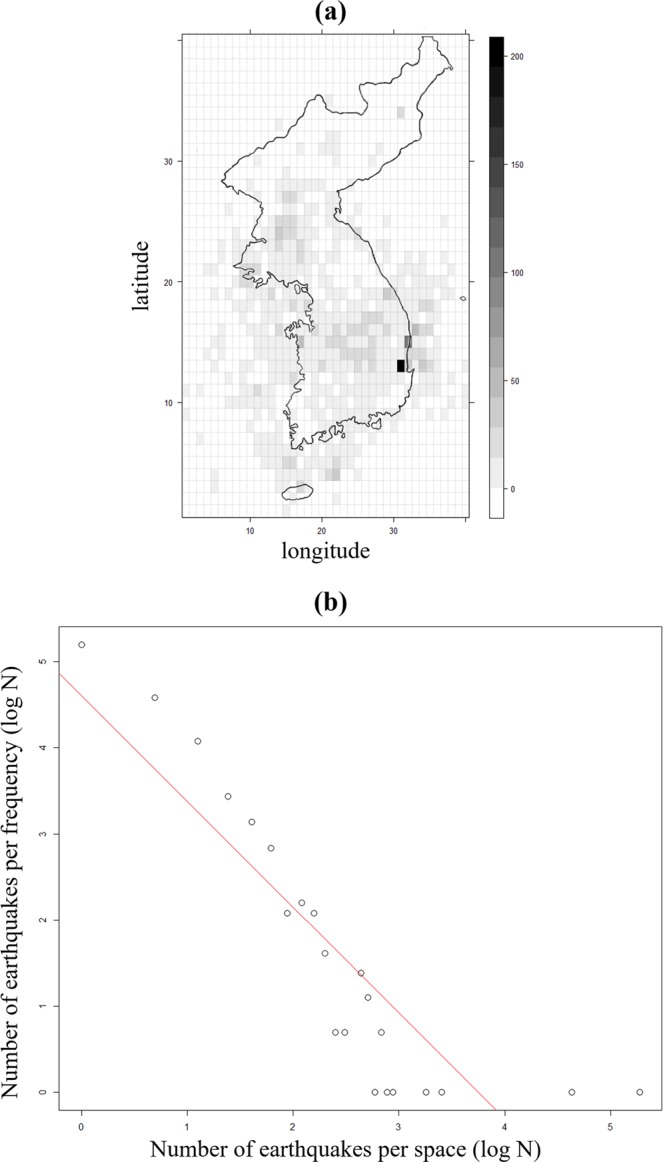
Visualization of earthquake activity in the Korean Peninsula in terms of spatial location and frequency of earthquakes per grid-section. (**a**) The entire Korean Peninsula is divided into a 40 × 40 rectangular grid and divided into spaces of the same size (top left: N43.00°, E122.8°; bottom right: N32.35°, E131.1°). The total size of the grid is about 674 km in width (longitude) and about 1184 km in length (latitude), and the size of each grid is 16.85 km in width and 29.6 km in length. Then, the frequencies of earthquakes were mapped to these grid-sections based on the longitude and latitude of the earthquakes observed in the Korean Peninsula earthquake measurement data. (**b**) This is a log-log plot showing the logarithmic regression of the frequency of earthquake occurrence (number of earthquakes per grid-section) and the number of grid-sections with that frequency of events for the 1600 sections. The x-axis represents the log of the number of earthquakes per grid-section, and the y-axis represents the log of the number of sections with the same earthquake frequency. For example, if seven grid sections had 10 earthquakes, the plotted x-axis value would be log(10) and the y-axis value would be log(7).

More than two-thirds of the Korean peninsula consist of granite and metamorphic rock. The granite mainly consists of Jurassic and Cretaceous granite, Daebo granite, and Bulguksa granite. Metamorphic rocks are mainly composed of gneiss and include shale, sandstone, and limestone. The primary sedimentary rocks are shale, sandstone, conglomerate, and limestone, and they are mainly distributed in the Gyeongsang Basin, including Gyeongsang-do Province. In the Cretaceous basins, including the Gyeongsang Basin, sediments, volcanic rocks, and tuff appear^43^. Although 1,733 earthquakes greater than ${M}_{L}$2 occurred in the Korean Peninsula, Fig. 1(a) shows that out of the 1600 grid-sections, 459 spaces experienced more than one earthquake, indicating the earthquakes did not occur spatially uniform. Most of the earthquakes observed in the Korean Peninsula can be identified to occur in either Gyeongsang-do Province in the southeast of the Korean Peninsula, or Hwanghae-do Province in the northwest. Gyeongju and Pohang, where the two strongest earthquakes occurred, particularly show frequent earthquake activities. A total of 535 earthquakes were identified in Gyeongsang-do Province, resulting in the highest frequency of earthquakes—31% of the total for the Peninsula. The logarithmic regression, coefficients of determination (*R*^2^), between the grid-section and the frequency of earthquakes was 0.7674, showing a high correlation between space and frequency. This suggests that the earthquake frequency of each grid-section and the number of grid sections follow a power law distribution. In other words, statistically, we prove that even if there are a large number of earthquakes, they do not occur spatially uniform, but rather tend to concentrate in specific regions.

### Analysis of earthquake activity in the Korean Peninsula considering temporal factors

To identify changes in the frequency of earthquakes by magnitude, the overall observation window 1978–2018, was divided into 10-year periods, and the establishment of the Gutenberg–Richter law was examined in each period and the total window.

**Figure 2:**
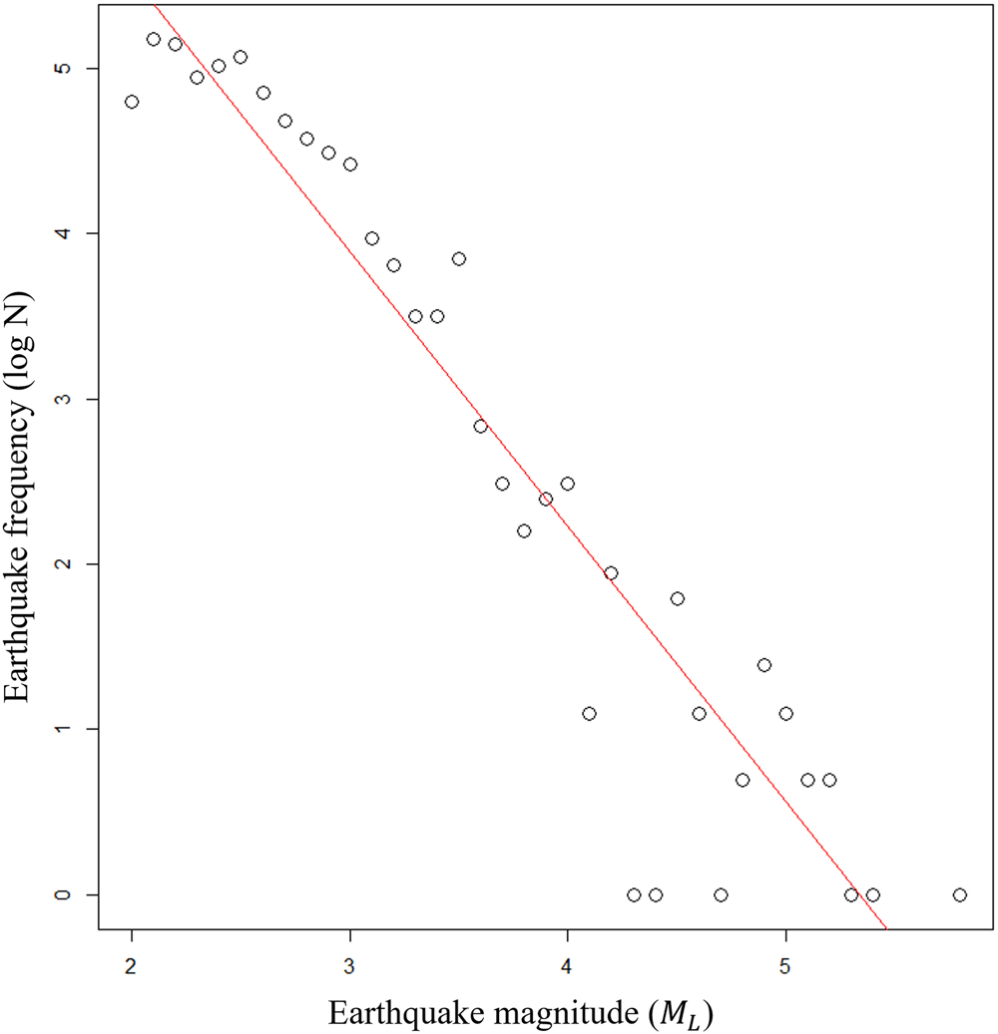
Visualization of the analytical result of the magnitude and frequency distribution of all earthquakes observed since earthquake observation began on the Korean Peninsula. Log-log plot between the magnitude and frequency of earthquakes observed from 1978 to 2018.

Figures 2 shows the result of the logarithmic regression between the magnitude and frequency of earthquakes throughout the entire period. The numerical values of the analyzed results are shown on Table 1. The fitness of the logarithmic-regression distribution for magnitude and frequency in the entire period was significant because the p-value was less than 0.0001. In other words, the Gutenberg-Richter law was established for the entire earthquake of the Korean Peninsula. After that, to confirm the establishment of the Gutenberg–Richter law by time, we moved the time window only in three-year steps and overlapped the time windows over the entire period. As a result of overlapping, the coefficient of determination of the power law distribution between the magnitude and frequency of earthquakes was less than 0.5 before 1998. In some cases, the p-value was higher than 0.05, and the Gutenberg-Richter law was not established. However, since 1998, the power law distributions consistently showed coefficients of determination above 0.5. The coefficients of determination from 1978 to 1997 and 1998 to 2018 were 0.6247 and 0.9025, respectively, indicating a significant difference between the periods. The reason for the large difference in the coefficients of determination of the power law between magnitude and frequency by period is that earthquakes before 1998 had a relatively low frequency. The fitness of the logarithmic regression model over the entire period was also significant, with a coefficient of determination of 0.9029, which showed a clear power law distribution between magnitude and frequency.

**Figure 3:**
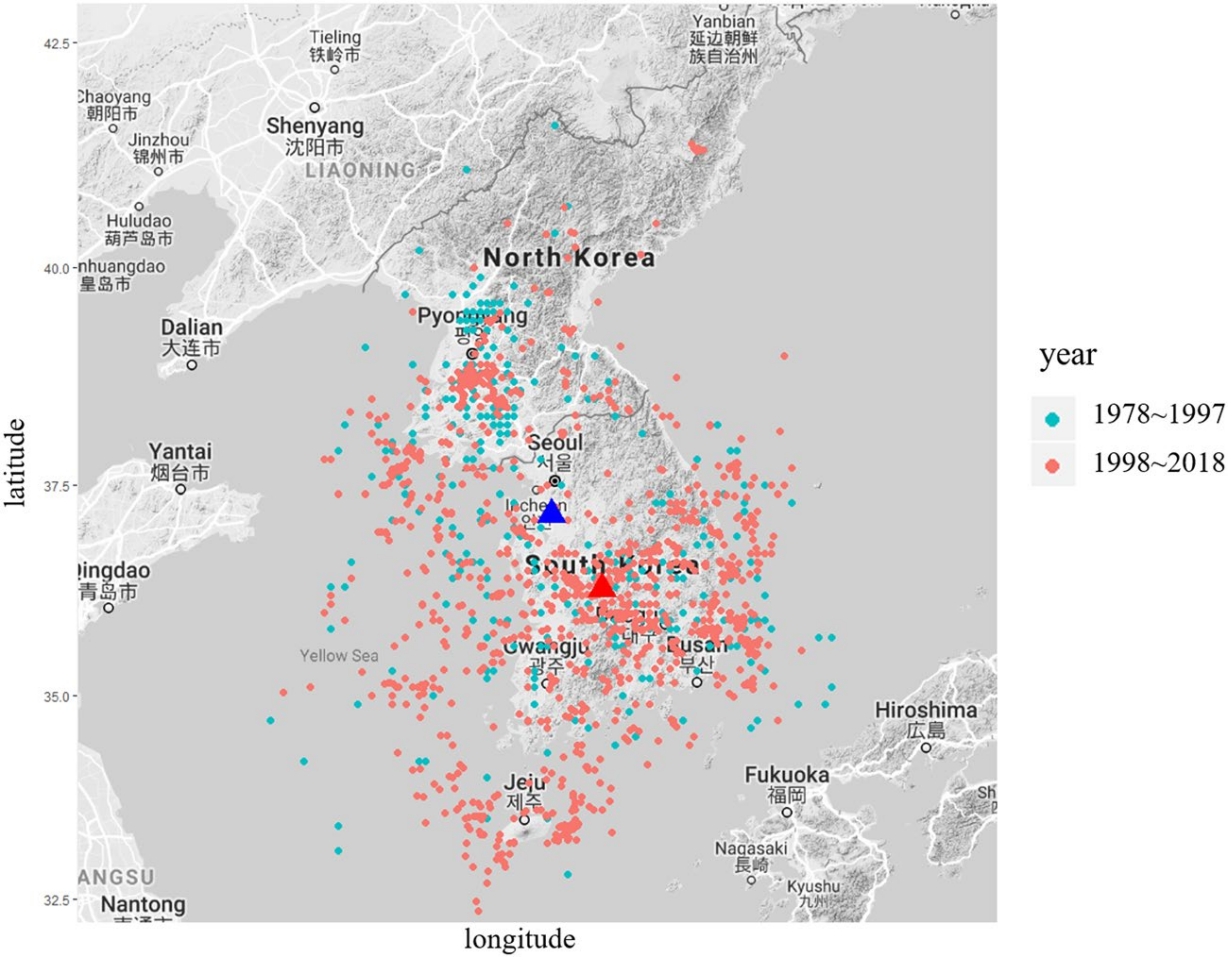
Visualization of earthquake occurrence in the periods before and after 1998. The blue and red triangles show the average latitude and longitude of all earthquake observations until 1997 and from 1998 to June 2018, respectively. The blue points indicate earthquakes before 1998 and red points indicate earthquakes after 1998. The map was drawn using the package ‘ggmap’ version 2.7.9 (https://cran.r-project.org/web/packages/ggmap/)^44^ in R version 3.5.1 (https://www.R-project.org/)^45^. The map of the Korea Peninsula was provided from the Google Maps (Map data^©^2019 Google) using the function ‘get_map’ in the ‘ggmap’ package.

**Table 1:** Results of the Logarithmic Regression by Period.

periods	${{\boldsymbol{R}}}^{2}$	F-statistic	p-value	periods	${{\boldsymbol{R}}}^{2}$	F-statistic	p-value
1978–1980	0.3898	11.5000	0.0032	1998–2000	0.5144	21.1900	0.0001
1979–1981	0.2146	4.3720	0.0528	1999–2001	0.6711	36.7300	<0.0001
1980–1982	0.4025	10.1000	0.0062	2000–2002	0.7967	74.4600	<0.0001
1981–1983	0.1128	2.1610	0.1598	2001–2003	0.8580	114.8000	<0.0001
1982–1984	0.1736	3.7820	0.0675	2002–2004	0.6568	38.2700	<0.0001
1983–1985	0.0935	1.6520	0.2170	2003–2005	0.6855	45.7800	<0.0001
1984–1986	0.2881	6.4750	0.0216	2004–2006	0.7274	50.6900	<0.0001
1985–1987	0.0827	1.3540	0.2628	2005–2007	0.6640	35.5800	<0.0001
1986–1988	0.1390	2.0980	0.1712	2006–2008	0.8133	69.7400	<0.0001
1987–1989	0.0673	0.5779	0.4690	2007–2009	0.7935	65.3300	<0.0001
1988–1990	0.0025	0.0277	0.8707	2008–2010	0.6723	34.8800	<0.0001
1989–1991	0.0368	0.4975	0.4931	2009–2011	0.5465	20.4900	<0.0001
1990–1992	0.0209	0.3423	0.5666	2010–2012	0.6823	36.5100	<0.0001
1991–1993	0.2771	6.5170	0.0205	2011–2013	0.7080	46.0600	<0.0001
1992–1994	0.3713	13.5800	0.0012	2012–2014	0.6734	43.3000	<0.0001
1993–1995	0.5077	22.6900	<0.0001	2013–2015	0.6621	41.1500	<0.0001
1994–1996	0.4381	16.3700	0.0005	2014–2016	0.7181	56.0300	<0.0001
1995–1997	0.2360	5.2500	0.0349	2015–2017	0.7968	90.1900	<0.0001
1996–1998	0.2750	7.5880	0.0122	2016–2018	0.8479	122.6000	<0.0001
1997–1999	0.2516	6.7220	0.0174	Entire period	0.9029	316.1000	<0.0001

Because there was a significant difference in the coefficient of determination of the power law, coefficients of determination from before and after 1998, a visualization, and further analysis of frequency and magnitude were established for these two periods.

Even though the earthquake activity observation period was divided in half, the earthquake activity record since 1998 accounted for about 80% of all earthquake activities. This is depicted in Fig. 3. From 1998–2018, seismic activity was concentrated in a small area in the southern part of the Korean Peninsula, mainly near Gyeongsang-do Province, confirming that the average location center of earthquakes has moved southeasterly. Table 2 shows the results of the t-test to determine the seismic changes in the Korean Peninsula over the two periods. Welch’s t-test, which is a heteroscedastic test, was performed because the two equal time variance assumptions did not hold during the t-test. The t value was found to be high at 13.782, indicating that there is a statistically significant difference in the magnitude of earthquakes between before and after 1998.

**Table 2:** Welch’s t-test of the magnitude of the earthquakes by period.

Year	t-value	Degree of freedom	p-value	Observation	Average magnitude
1978–1997	13.782	479.43	2.2e-16	348	3.0143
1998–2018				1385	2.5453

## Discussion

In this study, the earthquake phenomena of the Korean Peninsula were analyzed in consideration of spatiotemporal factors. To take into account spatial factors, the Korean Peninsula was divided into 1,600 equal sections using a grid. The logarithmic regression then verified that the relation between the grid section where the earthquake occurred and the frequency(number) of earthquakes in that grid-section follows the power law distribution. In another analysis, the earthquake observation period was divided to consider the temporal factors and to determine whether or not the Gutenberg–Richter law was established by period. As a result, earthquakes occurring after 1998 showed 1.7372 times higher coefficients of determination of the power law distribution than before, and the t-test confirmed that the magnitude of earthquakes occurring after 1998 was smaller than of those occurring earlier. In other words, according to the power law distribution between the grid section where the earthquake occurred and the frequency of earthquakes in that grid-section, the earthquakes are concentrated in specific regions. Hence, it was confirmed that many earthquakes occurred in Gyeongsang-do Province. In addition, since 1998, the power law distribution between the magnitude and frequency of earthquakes had a high coefficient of determination, and the average number of earthquakes has increased significantly. This increase in the number of earthquakes resulted in a number of small-scale earthquakes; also a few large-scale earthquakes occurred. In particular, since 1998, five out of seven earthquakes of ${M}_{L}$5 or higher have occurred, and 80% of the total earthquakes occurred. Moreover, Due to the concentration of earthquakes in specific regions, earthquakes of ${M}_{L}$5 or higher also occurred five times in Gyeongsang-do Province alone. As a result, large-scale earthquakes can occur at any time in specific regions in which earthquakes are concentrated.

The earthquake spatial data of this study were generated in rectangular grid-sections based on latitude and longitude. This is different from previous studies that have formed earthquake networks. This study presents ideas from the spatial point of view of the earthquakes. We argue that follow-up studies need to consider spatial factors due to the concentration of earthquakes in specific regions. Moreover, we revealed that the pattern of earthquakes differs significantly over time windows, which may be useful for earthquake-related social policymaking and follow-up studies.

## Materials and Methods

This study used the earthquake magnitude and location data of the Korean Peninsula from 1978 to June 2018 from the Weather Data Open Portal of the Korea Meteorological Administration. There is a total of 264 earthquake measuring stations in the Republic of Korea. The types of stations are classified into 95 broadband seismometers, 27 short-period seismometers, and 142 accelerometers. The collected data includes the date, magnitude, epicenter, and latitude and longitude of the earthquakes. Of the 4,107 datasets, 31 datasets with missing information on location or magnitude of earthquakes were removed. In addition, since the earthquake data from 1978 to 1998 were collected by analog observations of earthquakes with a magnitude greater than ${M}_{L}$2, only datasets of events of ${M}_{L}$2 or higher were used after 1999, when digital observation was implemented. The statistical lower limit for earthquakes on the Korean Peninsula suggested by the Korea Meteorological Administration is more than ${M}_{L}$2. Therefore, we excluded micro-earthquakes of less than ${M}_{L}$2 from the dataset. After such filtering, a total of 1,733 datasets were available and used for this study.

Table 3 lists the descriptive statistics of earthquakes that occurred on the Korean Peninsula, including the standard deviation (S.D.) for each value. As only earthquakes of ${M}_{L}$2 or higher were used, the minimum earthquake magnitude value was ${M}_{L}$2, the average value ${M}_{L}\,$2.64, and the maximum value ${M}_{L}$5.8. The latitude of the earthquake region ranged from N32.35° to N41.60° and the longitude ranged from E122.8° to E131.1°, covering the entire Korean Peninsula.

**Table 3:** Descriptive Statistics of the Korean Peninsula Seismic Data.

Parameter	Min.	Median	Mean	Max.	S.D.
Magnitude (${{\boldsymbol{M}}}_{{\boldsymbol{L}}}$)	2.0	2.5	2.64	5.8	1.4
Latitude (90° N)	32.35	36.14	36.46	41.6	2.98
Longitude (180° E)	122.8	127.7	127.6	131.1	2.82

**Figure 4:**
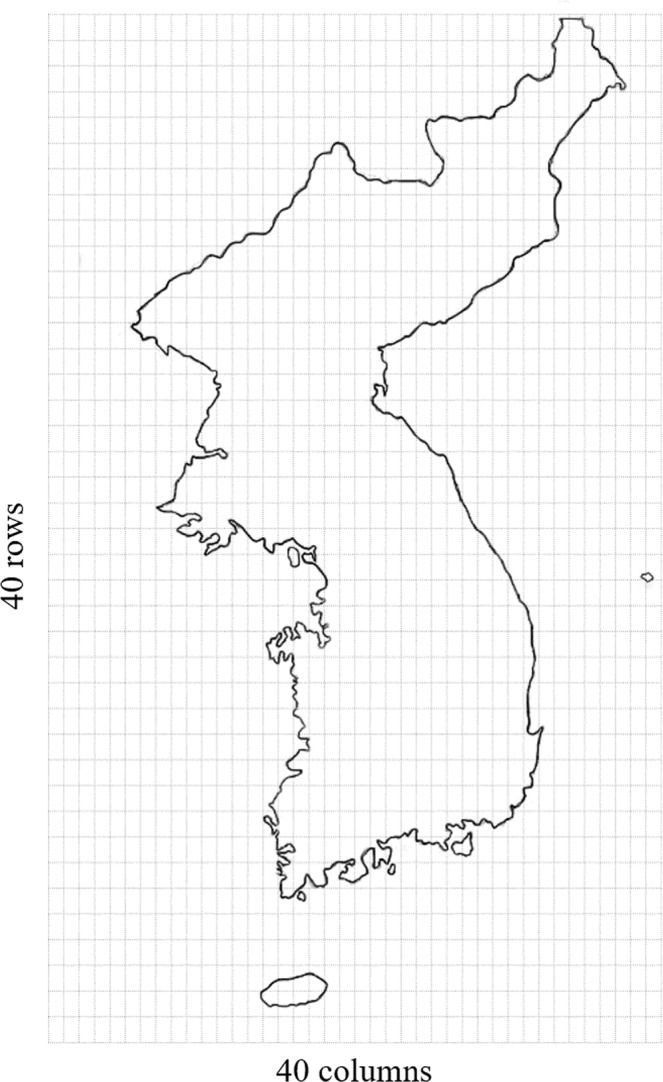
An image of the entire Korean Peninsula, defining its latitude as N32.35° to N43.00° and longitude as E122.8° to E131.1°, then divided into a uniform grid with 40 rows and 40 columns, for a total of 1,600 sections.

To consider spatial factors, the entire area of the Korean Peninsula was divided into a uniform grid with 40 rows and 40 columns. This is depicted in Fig. 4. First, this study analyzed the frequency of earthquake occurrence in each grid-section followed by mapping the frequency of earthquake occurrences in various grid sections. Moreover, this study considered the relationship between the magnitude and frequency of earthquakes by period.

The distribution of the frequency of earthquakes in each space and the number of spaces corresponding to a specific number of events were confirmed by logarithmic regression (Eq. 1). The dependent variable was the number of grid sections in which earthquakes occurred, and the independent variable was the frequency (total number) of earthquakes in a grid section. Sections where no earthquake occurred were removed, and only the sections where at least one earthquake occurred were used. Second, the analysis of magnitude and frequency to confirm the establishment of the Gutenberg–Richter law by period was conducted in the same way as the spatial analysis except that the independent variable was the magnitude of the earthquake and the dependent variable was the frequency of earthquakes by time windows. Finally, a t-test was performed to determine any overall change in magnitude of the earthquakes over time. The stats package in R version 3.5.1 was used for all analysis.

The logarithmic transformed regression and t-test equations used in the two analyses are as follows:

1 $$\log \,{y}_{i}=\alpha +\beta \,\log ({x}_{i})+{\varepsilon }_{i}\,(i=1\,to\,n)$$

2 $$t=\frac{\overline{{X}_{A}}-\overline{{X}_{B}}}{{S}_{A-B}}$$

In Eq. (1), *y* is a dependent variable and *x* is an independent variable. α*i* s a regression coefficient for estimating the *y* intercept, *ε* is the error term, and *n* is the number of data. *β* is a coefficient that reflects the influence of log(*x*) and measures the change rate of *y* according to the fine change rate of *x*. In Eq. (2), t is the average difference of the two groups by the data deviation calculated from the two groups. *A* and *B* denote each group, *S* is the standard deviation, and $\bar{X}$ is the average.
